# Immunotherapy as a Therapeutic Strategy for Gastrointestinal Cancer—Current Treatment Options and Future Perspectives

**DOI:** 10.3390/ijms23126664

**Published:** 2022-06-15

**Authors:** Evangelos Koustas, Eleni-Myrto Trifylli, Panagiotis Sarantis, Nikolaos Papadopoulos, Eleni Karapedi, Georgios Aloizos, Christos Damaskos, Nikolaos Garmpis, Anna Garmpi, Kostas A. Papavassiliou, Michalis V. Karamouzis, Athanasios G. Papavassiliou

**Affiliations:** 1 Department of Biological Chemistry, Medical School, National and Kapodistrian University of Athens, 11527 Athens, Greece; vang.koustas@gmail.com (E.K.); trif.lena@gmail.com (E.-M.T.); panayotissarantis@gmail.com (P.S.); konpapav@med.uoa.gr (K.A.P.); 2 First Department of Internal Medicine, 417 Army Share Fund Hospital, 11521 Athens, Greece; npnck7@yahoo.com (N.P.); karapedi.eleni@gmail.com (E.K.); aloizosgio@yahoo.gr (G.A.); 3 ‘N.S. Christeas’ Laboratory of Experimental Surgery and Surgical Research, Medical School, National and Kapodistrian University of Athens, 11527 Athens, Greece; x_damaskos@yahoo.gr; 4 Renal Transplantation Unit, ‘Laiko’ General Hospital, 11527 Athens, Greece; 5 Second Department of Propaedeutic Surgery, ‘Laiko’ General Hospital, Medical School, National and Kapodistrian University of Athens, 11527 Athens, Greece; nikosg22@hotmail.com; 6 First Department of Pathology, Medical School, National and Kapodistrian University of Athens, 11527 Athens, Greece; annagar@windowslive.com

**Keywords:** gastrointestinal tumors, cancer, immunotherapy, checkpoint inhibitors, cancer vaccine, tumor microenvironment

## Abstract

Gastrointestinal (GI) cancer constitutes a highly lethal entity among malignancies in the last decades and is still a major challenge for cancer therapeutic options. Despite the current combinational treatment strategies, including chemotherapy, surgery, radiotherapy, and targeted therapies, the survival rates remain notably low for patients with advanced disease. A better knowledge of the molecular mechanisms that influence tumor progression and the development of optimal therapeutic strategies for GI malignancies are urgently needed. Currently, the development and the assessment of the efficacy of immunotherapeutic agents in GI cancer are in the spotlight of several clinical trials. Thus, several new modalities and combinational treatments with other anti-neoplastic agents have been identified and evaluated for their efficiency in cancer management, including immune checkpoint inhibitors, adoptive cell transfer, chimeric antigen receptor (CAR)-T cell therapy, cancer vaccines, and/or combinations thereof. Understanding the interrelation among the tumor microenvironment, cancer progression, and immune resistance is pivotal for the optimal therapeutic management of all gastrointestinal solid tumors. This review will shed light on the recent advances and future directions of immunotherapy for malignant tumors of the GI system.

## 1. Introduction

There is a global trend of a continuously increasing incidence of gastrointestinal (GI) cancers with various epidemiological backgrounds and genetic and epigenetic aberrations, making them the most frequent cancers globally with generally high mortality rates. Despite various conventional therapeutic options, such as chemotherapy, radiotherapy, and surgical approaches, patients with end-stage disease present an unfavorable prognosis [1]. As a result, novel anti-cancer therapies have been developed, such as immunotherapy.

Immunotherapy is considered a step-up strategy for the management of a wide spectrum of malignancies, especially when those that have reached their end stage. Immunotherapeutic agents exhibit a favorable targeted effect on malignant cells, either promoting or inhibiting immune responses via interacting with immunogens presented on malignant cells. Importantly, they do not induce any detrimental effects on normal, non-cancerous cells, which makes them an optimal therapeutic option [2].

There is a wide range of immunotherapeutic modalities that are introduced in GI cancer management, including immune checkpoint inhibitors, adoptive cell transfer, chimeric antigen receptor (CAR)-T cell therapy, cancer vaccines, and/or combinations thereof. The least beneficial effect for cancer management is demonstrated by the newly developed cancer vaccines [3], in comparison with immune checkpoint inhibitors that present remarkable effects. Immune checkpoint blockade constitutes one of the most widely used immunotherapeutic modalities, aiming at three significant molecular targets: (i) the programmed death-ligand 1 (PD-L1), presented on the surface of cancer cells and antigen-presenting cells (APCs), (ii) the programmed cell death protein (PD-1) on the surface of lymphocytes, and (iii) the cytotoxic T-lymphocyte associated protein-4 (CTLA-4) on the surface of regulatory T cells (Tregs). All of these molecular targets have a pivotal role in cancer-related immune responses, due to the fact that they induce the so-called tumor escape mechanism, avoiding the normal cellular apoptotic process and, therefore, leading to the immortalization of cancer cells [4].

A better knowledge of the mechanisms that lead to tumor escape as well as a better understanding of the impact of tumor microenvironment (TME) on the efficacy of immunotherapy, are considered crucial for the optimal management of GI cancer. There is an important interplay between immune responses and TME, being highly investigated especially for gastric and colorectal malignancies [5], as it provides multiple potential therapeutic targets for anti-neoplastic treatment [6].

**Figure 1:**
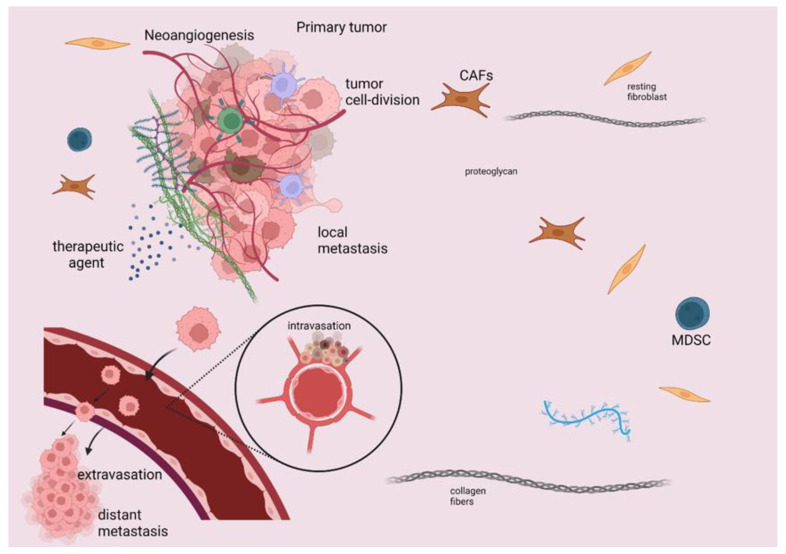
Schematic presentation of TME elements that induce immunosuppression, tumor progression, and metastasis. TME constitutes a surrounding stroma with a wide variety of cells, such as immune cells, fibroblasts, as well as many regulatory molecules, which are considered potential druggable targets. MDSC, B and T regulatory cells, TAMs, and cancer-associated fibroblasts (CAFs) have quite significant implications for cancer management, as they elicit an immunosuppressive effect that limits the efficacy of immunotherapeutic agents. TME immunosuppression is attributed to various molecules secreted by cancer cells, such as inhibitory checkpoints leading to the recruitment of immune cells, including MDSCs, T regulatory cells, and TAMs. This figure was created with BioRender.com, accessed on 14 May 2022 (agreement number UO23X0OEMQ).

The immune response is considered a multiplex and multi-stepped procedure, comprising three distinct steps: the first asymptomatic step includes the primary endeavor of cancer cells for elimination, whereby innate immune cells recognize the malignant cells and try to eliminate them via their cytotoxic effect and the production of antibodies against immunogens on the malignant cell surface through the adaptive immune response. This first step is followed by the step of balance, during which limitation of tumor progression is unfeasible, since malignant tumors can escape immunosurveillance. The last step, the tumor escape step, is symptomatic and takes place when the tumor progresses despite immunotherapy [7,8,9]. This process is achieved by tumor cells as they escape from innate immune system recognition. The restriction of antigen expression on cancer cell surfaces is considered the key mechanism for avoiding recognition by CD8+ T cells [10]. The induction of TME immunosuppression is attributed to many molecules secreted by cancer cells, such as inhibitory checkpoints that lead to the recruitment of immune cells. Among these recruited immune cells are myeloid-derived suppressor cells (MDSCs), which are associated with poor prognosis [11] and immune resistance [12,13]. Some other immunosuppressive cells are T and B regulatory cells, as well as tumor-associated macrophages (TAMs), which also promote tumor progression and neoangiogenesis [14]. Specifically, Bregs impede the action of cytotoxic T cells and promote carcinogenesis mainly via the secretion of Il-10, while Tregs express fork head box P3 (FOXP3) and they are associated with the downregulation or suppression of T-effector cells [12,15]. In Figure 1, we present some of the main components of TME.

The above phenomena imply the significant influence of TME on tumor progression, invasion, metastasis, and development of therapy resistance attributed to the impediment of cytotoxic T cells’ action, as well as the secretion of immunosuppressive molecules that interfere with immune anti-cancer responses [16,17].

**Figure 2:**
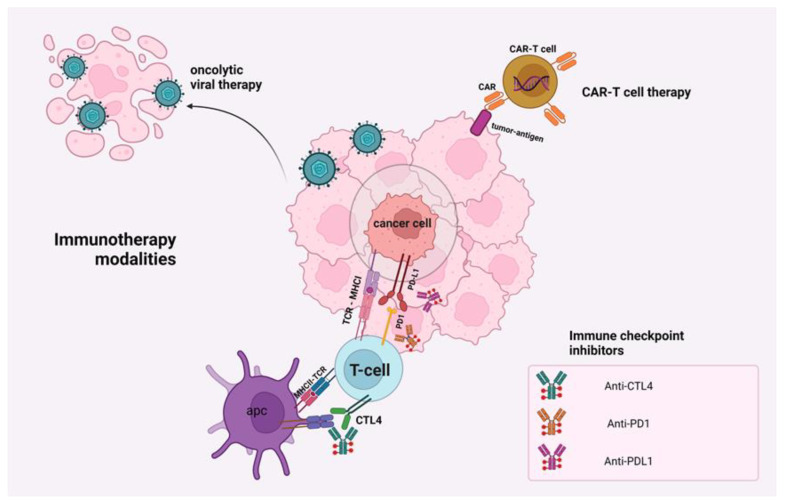
Schematic presentation of immunotherapy modalities and their associated targets. There is a wide range of immunotherapeutic modalities that are introduced in the GI cancer management, including immune checkpoint inhibitors, adoptive cell transfer, chimeric antigen receptor (CAR)-T cell therapy, cancer vaccines, and/or combinations of all the aforementioned. This figure was created with BioRender.com (agreement number HP23X0I0W3).

Additionally, the expression of inhibitory immune checkpoints enhances the immunosuppressive mechanism of tumor cells. Figure 2 depicts these checkpoints on tumor cells and the action of PD1/PD-L1 and CTLA-1/B7, which constitute important therapeutic targets. While under normal conditions, immune checkpoints on T cell surfaces act as a brake for triggering an immune response, tumor cells hijack these molecules to promote the tumor-escape phenomenon [18]. These immune checkpoints, including CTLA-4, PD-1, and PD-L1, are involved in the biology of many malignant tumors [19]. However, there are many reports on tumors displaying immunotherapy resistance, and as a result, there is no evident improvement in survival rate [18,20].

Herein, we review the application of immunotherapy in some of the main types of GI malignancies, such as gastric cancer (GC), pancreatic adenocarcinoma (PAC), as well as colorectal cancers (CRCs), hepatocellular carcinoma (HCC), and cholangiocarcinoma (CCA).

## 2. Mechanisms of Action of Immunotherapeutic Modalities

The immune system has a significant impact on tumor progression and therapeutic efficacy. It can be modulated either in an active or a passive manner. The former approach utilizes vaccines, which trigger an anti-cancer immune response via presenting tumoral antigens to immune cells in order to eliminate the malignant cells, while the latter approach includes the administration of immune cells and antibody-based treatment. Immune checkpoint inhibitors are monoclonal antibodies (mAbs) that interact with PD-1, as well as CTL-4 with concomitant stimulation of T cells (effector). These mAbs block the proteins on the surface of T-cells or on the surface of cancer cells. The stimulation of PD-L1, PD-1, and CTLA-4 proteins on T cells impedes the recognition of cancer cells and prevents their elimination. Blocking these proteins permits the activation of T-cells and the destruction of cancer cells, as well as the control of tumor growth and recurrence. The mechanism of action of cancer vaccines includes the stimulation of T-cells through the administration of messenger RNA (mRNA) or other types of vectors (viral, bacterial, or yeast-based vaccines). Another modality is CAR-T cell treatment, based on genetically engineered T cells of the patient, whereby a gene responsible for the production of the CAR protein is inserted in the cells’ genome. After the CAR-T cells are infused in the patient, the extracellular antigen recognition domain (ectodomain) of chimeric antigen receptor (single-chain Fragment variant (scFv)) interacts with the neoantigens or other proteins on the surface of malignant cells [21,22,23]. Thus far, there are five generations of CAR-T cells which are differentiated by the intracellular signaling domain [23]. The (i) first generation of CAR-T cells include a ζ-chain intracellular domain that does not present a sufficient response, while there is a need for exogenous IL-2 supplementation. The (ii) second generation of CAR-T cells include OX-40, or CD28, and 4-1BB intracellular domains. The latter can be activated in the absence of the antigen, a phenomenon called constitutive stimulation. The (iii) third generation combines various costimulatory signaling domains with similar effects to the second generation. The (iv) fourth generation comprises the so-called “TRUCK” T cells that include a cytokine, such as IL-12, which leads to a cytokine-mediated killing of cancer cells via the attraction and the activation of additional immune cells, while the (v) fifth generation includes IL-2 receptors that induce JAK/STAT pathway activation [24,25]. Additionally, there are mAbs with a scFv that interact with T-cells (effector) and prevent the stimulation of cytokines, which either induces or suppresses the immunological response. Furthermore, the antibody-based therapy represents a promising therapeutic modality for the management of solid tumors. The introduction of bi- and tri-specific antibodies is considered a revolutionary approach. The former includes one arm specialized for CD3 and a second arm targets the neoantigen, while the stimulation of T cells is achieved without the involvement of MHC [21,26]. Finally, there is another immunotherapy modality based on the depletion of regulatory T cells (Treg), for example daclizumab, a monoclonal antibody against CD25 of Tregs [27].

## 3. Immunotherapy in Gastrointestinal Cancers

Conventionally, the mainstay treatment for GI cancers has been focused around radiation and/or chemotherapy, antiangiogenic therapy, surgical intervention, or a combination of these treatments; nevertheless, the overall survival of patients with GI tumors remains poor. Aiming to decrease morbidity and improve mortality outcomes in GI cancer patients, trends have shifted over the last couple of decades towards the use of new agents which re-activate the immune system, namely, immunotherapy. For GI cancers, immunotherapy consists mainly of ICI, cytokine, adoptive cell treatment, and vaccine therapies.

## 4. Immunotherapy in Pancreatic Adenocarcinoma

Pancreatic adenocarcinoma (PAC) is considered a lethal disease worldwide with a dismal prognosis and low five year-survival rate (<10%) [28,29], while it constituted the third most common cancer-related cause of death in the US in 2019. Diagnosis is common in the late stages of cancer when tumor-resection is already unfeasible, due to early disseminated metastatic disease and the ineffective anti-neoplastic treatments for this malignancy [30]. The risk of PAC occurrence is correlated with advanced age, with infrequent cases of patients below 40 years old [31]. Major risk factors for PAC development are tobacco abuse, chronic alcoholism, obesity (body mass index (BMI) value >30 kg/m^2^), long-lasting Diabetes mellitus (DM), increasing the risk of pancreatic carcinogenesis up to 2.4 times, as well as chronic pancreatitis, which doubles the risk for pancreatic carcinogenesis [32]. Reduced risk of PAC (25%) development is reported in some individuals that present allergies, probably due to the overactive immune responses that contribute to an anti-cancer effect [33]. PAC exhibits many genetic and epigenetic aberrations, including the novel mutations of *TP53*, *KRAS*, the mutant *ERBB2*, *CDKN2A*, and *BRCA2* genes, as well as the deletion mutations of the *DPC4* gene. Overall, the variety of gene mutations and the combination of the upregulation of growth factors, such as interleukins 8, 6, and 1, vascular endothelial growth factor (VEGF), as well as Tumor necrosis factor (TNF), make pancreatic cancer remarkably unsusceptible to treatment [34]. The anti-tumor immune response is elicited by the appearance of chronic inflammation, which is closely correlated with carcinogenesis. Promoters of cellular growth, such as Fibroblast activation protein (FAP), cancer-associated fibroblasts (CAFs), and fibronectin, induce the activation of the mechanistic target of rapamycin (mTOR) and the secretion of cytokines, such as IL6 and CXCL12, which recruit T-cells CD4+ [35,36]. Immunotherapy is a recent breakthrough anti-neoplastic treatment strategy that was added to PAC management on the backbone of the conventional anti-cancer treatment strategies. The PAC management starts with the surgical treatment, followed by chemoradiation, and then the immune modulation therapy is applied [37]. Major obstacles to immunotherapy efficacy are the appearance of stromal desmoplasia [38], a higher amount of collagen type I and IV fibers that impede stromal T-cells from interacting with malignant cells, and thus, promote PDAC cell growth, expansion, and immunotherapy resistance [39]. Moreover, the tumor microenvironment (TME) contains tumor-infiltrating lymphocytes (TILs) that are associated with immunotherapy resistance and poor prognosis [40]. Studies related to the efficacy of neoadjuvant or adjuvant immunotherapy show no significant variation in the OS, while neoadjuvant immunotherapy proved beneficial. Particularly, neoadjuvant immunotherapy prevents recurrence via limiting post-surgical immunosuppression [41].

### 4.1. Immune Checkpoints Inhibition in PAC

There are many clinical trials for assessing immune checkpoint inhibitors in PAC. However, the effectiveness of these agents proved limited based on phase I and II data due to the lack of antigenicity [42]. The targets for these agents include CTLA-4, PD-1, and PD-L1, which are involved in many malignant tumors [43]. Based on phase Ib/II (NCT02305186), the combinational treatment consisting of Pembrolizumab, a PD-1 inhibitor (IV administration of 200 mg, every 3 weeks), radiotherapy, and capecitabine, a cytotoxic agent (oral administration of 825 mg/m^2^ 1×2 per day) proved beneficial as a neoadjuvant therapeutic strategy with a notable increase of overall survival (OS) compared to radiation alone [44]. For resectable PDAC, there is an ongoing phase II trial (NCT03727880) assessing the combination of Pembrolizumab with defactinib (a focal adhesion kinase inhibitor) or Pembrolizumab as an adjuvant or neoadjuvant immunotherapeutic strategy for resectable tumors [45]. For advanced disease or metastatic PDAC, there is a phaseIb/II trial (NCT02331251) assessing the combinational treatment with gemcitabine, Pembrolizumab, and nab-paclitaxel. The doses were based on whether chemotherapy was previously administered or not. For the first category of patients, the doses were modified, including nab-paclitaxel (100 mg/m^2^ every 21 days on days 1 and 8) andgemcitabine 800 mg/m^2^, in comparison with the groups of patients who did not have a history of previous chemotherapy, with the former being 125 mg/m^2^ every 21 days on days 1 and 8 and the latter 1000 mg/m^2^. Meanwhile, the Pembrolizumab was given intravenously before chemotherapy at a dose of 2 mg/kg/every 21 days, administered for more than 30 min [46]. Another phase II study (NCT02527434) of the combinational treatment of Tremelimumab, a CTLA-4 inhibitor (15 mg/kg), with gemcitabine (28 cycles of 1000 mg/m^2^ on days 1, 8, and 15) [42,47] or durvalumab (a PD-1 inhibitor) demonstrated moderate favorable effects in comparison to the single-agent treatment either with durvalumab or Tremelimumab. Furthermore, a phase III study (NCT03977272) compares the therapeutic effects of mFOLFIRINOX as monotherapy with the combinational treatment of mFOLFIRINOX and camrelizumab (a PD-1 inhibitor) [48]. Some other ICIs that are currently studied are sintilimab (a PD-1 inhibitor) and Toripalimab (a PD-L1 inhibitor). The latter is studied in a phase Ib/II trial including the combination treatment of toripalimad, gemcitabine, and nab-paclitaxel [42]. Additionally, there are various clinical trials that assess the efficacy of ipilimumab, a CTL4-4 inhibitor, in advanced metastatic PDAC, such as the phase Ib trial (NCT01473940), which combines gemcitabine (1000 mg/m^2^) and ipilimumab (3 mg/kg), demonstrating that the combination of the two agents had similar effects to gemcitabine monotherapy [49].

### 4.2. Oncolytic Viral Therapy in PAC

A combinational treatment of immune checkpoints blockade with viral cancer vaccines in PAC, such as adeno-associated viruses (AVV) with an anti-PD-1 agent, such aspembrolizumab, did not provide beneficial results. Some other viruses that are used are Herpes Simplex Virus-1 and 2 (HSV-1 and HSV-2), HSV1716, R3616vaccinia virus, as well as rabbit-MYXV poxvirus [50]. There are multiple clinical trials that assess the oncolytic viral treatment in PAC based on HSV and Adenovirus, such as a phase I clinical trial (NCT00638612) that assesses the intratumoral administration of AdV-Tk in resectable, unresectable, or locally advanced tumors (LAPC), a phase I/II clinical trial (NCT03225989) that assesses the intratumoral injection of LOAd703 in LAPC, as well as phase I trials for CAdVEC (NCT03740256) and Ad5-DS (NCT00638612, NCT00415454) for locally advanced tumors [51].

### 4.3. Cancer Vaccines in PAC

There are multiple combinational treatments of ICIs with different types of cancer vaccines, such as Peptide vaccines, CRS-207, or GVAX in combination with nivolumab and ipilimumab. Antigen-based cancer vaccines are also used, such as CV301, which targets mucin-1 (MUC-1) and carcinoembryonic antigen (CEA) 2, two major antigens in malignant pancreatic tumors [52]. Some other targets in pancreatic malignant cells include mKRAS, mesothelin, telomerase, and gastrin Mucin-1 protein, which participate in oncogenic signaling pathways that promote PAC progression and metastatic dissemination. Their inhibition could potentially induce a notable improvement in the overall survival of advanced cases [53]. Moreover, GVAX is a vaccine that is used in patients who have already undergone surgery in combination with chemoradiotherapy, while they are based on whole-cancer cells, which are designed to exhibit granulocyte-monocyte-colony stimulating factor (GM-CSF) [54]. A phase II study of GVAX combined with ipilimumab as a maintenance treatment in metastatic PDAC was not proven to be superior to chemotherapy [54]. KrasG12D mutation is considered the most common mutation in PAC (90% of the cases) and is targeted by the mKRAS vaccine [55]. Furthermore, antigen-presenting cells (APCs)-based vaccines, such as dendritic cells, induce T-cell activation and an anti-cancer immune reaction against a carcinoembryonic antigen as well as the telomerase reverse transcriptase [56]. Vaccines can also be based on bacteria, viruses, and yeasts as vectors, including vaccinia virus (VV), AVVs, alphaviruses, adenovirus (adV), bacilli Calmette–Guerin, *L. monocytogenes* that express mesothelin, such asthe pancreatic cells [57], and S. cerevisiae for GI-4000 vaccination, including four distinct vaccines [58].

### 4.4. Adoptive Cell Therapy in PAC

Chimeric antigen receptor (CAR) T cells constitute an adoptive cell treatment (ACT) designed to recognize specific antigens in malignant cells [59] and then induce their lysis. Some targetable antigens are mucin-1 (MUC1), mesothelin (MSLN), and CEA, while CART cells against the tumor-specific antigen (TSA) are currently under study [60]. Particularly, there are ongoing studies, such as the phase I trial on animal models (murine) with the MSLN-targeted CAR T-cell that proved to be beneficial and well-tolerated for PDAC patients. Another phase I study assessing the CD133-targeted CAR-T cell therapy was beneficial for a limited number of patients. Moreover, HER-2 and EGFR-targeted CAR-T cell therapies demonstrated toxicity not only for tumor cells but also for normal cells that shared the antigen (on-Target, off-Tumor Toxicity). Unfortunately, CAR-T cells are currently approved only for hematological malignancies [61].

### 4.5. Other Treatments That Target the TME Components

Inhibition of mTOR with agents such as SOM230, a somatostatin analog (pasireotide) or somatostatin (STT), or the use of PEGylated recombinant human hyaluronidase PH20 (PEGPH20) could overcome immune resistance attributed to stromal desmoplasia. However, SOM230 and STT proved unsuccessful [62,63]. PEGPH20, an inducer of hyaluronic acid breakdown, is associated with an aggressive phenotype, dismal prognosis, and induces T-cell recruitment [64], while it also increases the drug amount that reaches the pancreatic stroma, leading to a significantly improved survival rate and a longer interval of clinical disease absence (5.7 months) [65]. The combination of PEGPH20 with either gemcitabine or nab-paclitaxel also shows beneficial results in PAC management [66], while FAK inhibition with or without gemcitabine limits the density of the pancreatic stroma and decreases PAC invasiveness and expansion [67].

### 4.6. Future Approaches for Treating PAC

TME cells secrete a variety of pro-inflammatory cytokines that interfere with anti-cancer immune response (VEGF), interleukins (IL-6, IL-12) [68], epidermal growth factor (EGF), as well as tumor necrosis factor-alpha (TNF-a) and indoleamine-2,3-dioxygenase (IDO) [69].

Based on recent pre-clinical trials, some experimental ICIs have been identified, such as T cell immunoglobulin and mucin domain-containing protein 3 (TIM3), Indoleamine 2,3-dioxygenase (IDO), and V-domain Ig suppressor of T cell activation (VISTA). TIM3 is reported to have a role of a ‘checkpoint’ receptor, while its inhibition promotes the effect of PD-1 blockade [70]. IDO constitutes an enzyme encoded by the IDO1 gene, which takes part in tryptophan metabolism and immune responses. Its action leads to the production of ATP and nicotinamide adenine dinucleotide (NAD) that are involved in tumor development and progression due to the activation of suppressor T cells and cytotoxic T-cell (CTL) suppression. Inactivation of the above enzyme is studied in clinical trials for disseminated metastatic PAC [71]. VISTA constitutes a newly identified immune checkpoint protein, while its inhibition via anti-VISTA antibodies is considered a potential therapeutic strategy [72]. Furthermore, in patients with microsatellite stable (MSS) PAC who do not respond to ICIs, there are studies using a continuous infusion (1 week) of AMD3100, a small-molecule inhibitor that acts as an inhibitor of CXCR4 and promotes the immune responses in the secondary lesions. This strategy is based on the fact that pancreatic cancer cells exhibit a “coat” of CXCL12 which stimulates CXCR4 and modulates the immune response [73]. Meanwhile, bispecific antibodies (BsAbs) are now in the spotlight as they can interact either with two different epitopes of the same targeted antigen or with two distinct antigenic targets. Some of the main targets are EGFR, HER 2 and 3, as well as Angiopoietin-2 (ANG-2), prostate-specific membrane antigen (PSMA), and Delta Like Canonical Notch Ligand 1 (DLL1). KN046 constitutes a novel bispecific antibody that inhibits CTLA-4, PD-1, as well as PD-L1. There is an ongoing phase II trial for non-resectable, metastatic, or LAPC that assesses KN0465mpk (every 2 weeks) in combination with gemcitabine (1000 mg/m^2^, on days 1, 8, and 15 every 4 weeks) and nab-paclitaxel (125 mg/m^2^, on days 1, 8, and 15, every 4 weeks) [74]. A phase I/II adoptive T cell trial assesses anti-EGFR x anti-CD3 bispecific antibody (3–8 infusions), which constitutes a potent therapeutic strategy with a beneficial effect on overall survival [75].

## 5. Current Immunotherapy in Gastric Cancer

Gastric cancer (GC) is considered another highly lethal GI cancer worldwide, presenting a higher predominance in the male gender [76]. The amplification of GC cases is attributed to various risk factors, with the major being chronic infection of gastric mucosal cells with *Helicobacter pylori* (*H. pylori*), leading to non-cardia GC development, whereas gastroesophageal reflux disease (GERD) and obesity for cardia-type GC, as well as medically induced causes, such as chronic use of Proton-pump inhibitors (PPIs) [77]. In addition, there are plenty of genetic alterations leading to gastric carcinogenesis, such as *TP53* gene mutation, which constitutes the most frequent aberration (40% of cases), *ARID1A* mutation, and *BRCA2* mutation [76]. Cancer-associated inherited syndromes leading to the minority of GC cases include *Catenin Alpha 1* (*CTNNA1*) gene mutation and *Cadherin 1* (*CDH1*) gene, as well as *Glutathione S-Transferase Mu 1* (*GSTM1*)-null mutation, found in Diffuse Gastric Cancer (HDGC) and Lynch syndrome respectively, while there are also notable epigenetic modifications that promote gastric carcinogenesis [78].

There are multiple types of GC based on a molecular basis, such as (i) positive GC for Epstein–Barr virus (EBV)(8% of the cases), which is because interferon-gamma (INFg) is produced as a response to chronic viral infection, which also has an anti-cancer function; (ii) 22% of GC cases present microsatellite instability (MSI), exhibiting a high amount of genomic alterations and multiple cancer-associated antigens; and (iii) gastric tumors that present chromosomal instability found in most of the GC cases (50%) [79]. The first two are associated with a better prognosis in comparison with the last subgroup due to the existence of more TILs in the TME or inside the tumor [80,81] and PD-L1 overexpression, and thus, providing a target for immunotherapy [82].

### 5.1. Immune Checkpoints Modulation in GC

The main target of immune checkpoint blockade is T-cell effector interaction with cancer cells. However, tumor antigenicity interferes with T-cell physiological function and multiplication. Therefore, the blockade of molecular structures expressed by cancer cells, such as PD-1, CTL4, and PD-L1, could limit tumor cell progression [83,84].

GCs present with a better prognosis when Natural Killer (NK) cells and T-cells, including CD8+, memory, and CD3+, are abundant in the malignant gastric tumors [12]. High PD-L1 expression in gastric tumors, which is associated with MSI- and EBV-positive GC molecular subtypes, is considered a positive predictive biomarker for the effectiveness of PD-L1 inhibitors, while its overexpression is correlated with lymph node dissemination and metastasis, leading to dismal prognosis [85,86,87] and reduced survival [88]. Moreover, PD-1 located on CD8+ T-cells in the malignant tumor binds to PD-L1/-L2, leading to T-cell dysfunction and impaired proliferation that results in intratumoral immunosuppression especially when PD-L1 is overexpressed [89]. Based on phase III clinical trials, combined treatment of PD-L1 and PD-1 inhibitors with CTL-4 inhibitors did not show remarkable clinical benefit compared to other malignant tumors, [90]. There are multiple clinical trials, such as KEYNOTE-590 and CHECKMATE-649, that assess the combination of pembrolizumab with chemotherapy and the combination of nivolumab (a PD-L1 inhibitor) with chemotherapy, respectively. Moreover, based on phase III KEYNOTE-811 study (NCT03615326), Her2 on tumor cells can be targeted with trastuzumab and combined with pembrolizumab (PD-1 inhibitor). There is a notable improvement with this combinational treatment, which is considered first-line for Her2 positive tumors [91]. Moreover, based on the phase III studyATTRACTION-02, nivolumab provided long-term benefits in the setting of advanced gastro-esophageal or gastric cancer. Lastly, pembrolizumab as monotherapy is considered a potential therapeutic strategy for advanced gastro-esophageal or gastric cancers that were previously treated [92,93].

### 5.2. Cancer Vaccines in GC

There is a polyclonal antibody stimulator (PAS)vaccination that targets gastrin, which is related to tumor progression and growth and limits gastric tumor progression and dissemination in mice, when it is either combined or not with a PD-1 inhibitor [94]. There is a new phase II study that assesses the combinational treatment of G17DT with 5-FU and cisplatin, as well as a phase I/II targeting VEGFR-1 and VEGFR-2 combined with cisplatin and S-1, which demonstrated significant improvement in cases of advanced, relapsed GC positive for HLA-A 2402 [95,96].

### 5.3. Future Approaches for Treating GC

CAR-T cells therapeutic strategies are in the spotlight for GC management. Some of the most significant targets for GC are: HER2, MUC1, and CEA, as well as other targets, such as mesothelin, epithelial cell adhesion molecule (EpCAM), folate receptor 1 (FOLR1), claudin 18.2 (CLDN 18.2), and natural-killer receptor group 2, member D (NKG2D). However, these CAR-T cell therapies may lead to unfavorable effects because malignant and physiological tissues share the same targeted molecules. Other novel therapeutic targets in the spotlight for GC management include actin-related protein 2/3 (ARP 2/3), neoantigens such as CA19-9, CA-72-4, as well as B7H6, neuropilin-1, and anion-exchanger 1. These targets could open the horizons for the development of novel therapeutic strategies in the future; however, further research is required [97].

## 6. Current Immunotherapy in Hepatocellular Carcinoma

Hepatocellular carcinoma (HCC) is considered one of the most frequent lethal cancers in the population and the primary cause of death in patients with cirrhosis [98]. Based on global epidemiologic data, major risk factors for HCC are chronic viral infection with hepatitis C virus (HCV), hepatitis B virus (HBV) [99], as well as exposure to aflatoxins and chronic inflammatory diseases, such as cholestatic, alcoholic, and non-alcoholic steatohepatitis (NASH), or other autoimmune disorders followed by cirrhosis that predisposes to HCC development [98]. In early stages, HCC management includes surgical resection, radio-ablation, as well as liver transplantation [100]; however, in advanced disease, when HCC is inoperable, immunotherapy is considered a treatment option with a moderate improvement of overall survival [101]. Some well-studied therapies for HCC are the two oral multi-kinase inhibitors, sorafenib, which moderately increases overall survival [102,103], and lenvatinib, presenting non-inferiority compared to sorafenib in the REFLECT-phase III study [104,105]. The former used to be the first-line treatment since 2007 until the development of the latter in 2018. However, in recent years, immunotherapy gained ground as a therapeutic modality in HCC management [106].

### 6.1. Immune Checkpoint Blockade for HCC

CheckMate 040 (NCT01658878) is a clinical trial which assessed the combinational treatment of nivolumab (1 mg/kg) with ipilimumab (3 mg/kg) every 3 weeks and later nivolumab (240 mg every 2 weeks) for advanced HCC patients, who were already treated with Sorafenib [107]. Combinational treatment has proved superior to single-agent treatment with nivolumab [108]. KEYNOTE-224 constitutes another phase II trial that assesses the use of pembrolizumab, a PD-1 inhibitor, presenting a favorable anti-cancer effect in end-stage cases. Pembrolizumab is another FDA-approved ICI for HCC, which is also considered a second-line systemic therapy for HCC [109]. Another phase II/III clinical trial is ORIENT-32, which assesses the combinational treatment of sintilimab (200 mg every 3 weeks), another PD-1 inhibitor, with Bevacizumab biosimilar (IBI305, 15 mg/kg every 3 weeks), an antibody against VEGF, for patients who were already treated with sorafenib. This combinational treatment could be a potential novel therapeutic strategy, significantly improving the OS and progression-free survival (PFS), in comparison with sorafenib [110]. Inhibition of PD-L1 via atezolizumab in combination with bevacizumab, an anti-angiogenic agent that targets VEGF, showed a favorable effect on overall survival and prolonged the free-of cancer period compared to sorafenib as monotherapy [111]. Based on research on the combinational treatment of bevacizumab and atezolizumab in inoperable HCC, survival was significantly improved 84.8% for six months and 67.2% for 12 months, while for sorafenib it was 72.2% and 54.6%, respectively. At the same time, the progression-free interval was better (around 6.8 months) for the combinational therapy of atezolizumab–bevacizumab compared to monotherapy with Sorafenib (around 4.3 months) [112]. Lastly, the phase III study (RATIONALE-301) evaluated tislelizumab in comparison with sorafenib, as first-line systemic therapy, with promising anti-cancer effect for HCC, while another phase 3 (LEAP-002) study assessed the combinational treatment of Pembrolizumab (IV infusion on day 1 of every 21-days cycle, for 24 months) and lenvatinib (oral administration of 12 or 8 mg for body weight ≥60 kg or <60 kg, respectively) as first-line systemic therapy for patients with advanced HCC [113]. Finally, there is the HIMALAYA phase III study that assessed the combination of tremelimumab with durvalumab and the single-treatment with durvalumab compared to sorafenib for advanced HCC patients, who did not receive any previous treatment, demonstrating favorable results for the combination of tremelimumab with durvalumab, compared with sorafenib alone, while durvalumab monotherapy proved non-inferior to sorafenib [114].

In Table 1, we present a summary of the results from clinical trials that assess the utilization of ICIs in HCC management.

### 6.2. Utilization of Oncolytic Viral Therapy in HCC

Oncolytic viral treatment has also demonstrated favorable anti-neoplastic effects for HCC. There are reports about this modality and the use of a viral vector, such as the Herpes simplex 1 (HSV-1) virus (the oncolytic ICP0-null virus (d0-GFP) such as in LCSOV, G47Δ, and HSV-1-T-01 types. However, these are still at a preclinical phase, while adenovirus and vaccinia virus are also used. Additionally, many other treatments are reported, including CNHK500, ONYX-015, AD, ZD55-IFN-β, and Smac/ZD55-TRAIL, which use adenovirus as a vector [112]. Meanwhile, vaccinia-based viral vectors are used in the JX-594 therapy (modified poxvirus), which is in phase II/III trials, as well as in CVV [112,115]. JX-594 therapy (also called pexastimo gene devacirep vec (Pexa-Vec)), which is based on an oncolytic modified poxvirus, is under clinical trials for HCC management, while there is a phase III PHOCUS trial (NCT02562755) which assesses the application of Pexa-Vec in combination with sorafenib versus the monotherapy with sorafenib. Moreover, there is the TRAVERSE phase IIb trial that assesses the Pexa-Vec in cases of advanced HCC patients after the therapeutic failure of sorafenib [106,116,117].

### 6.3. Cancer Vaccine in HCC Management

There are multiple vaccine-based immunotherapeutic agents which have been assessed in the HEPAVAC project [118]. There are studies using dendritic cell vaccine (DC vaccine) combined with a PD-L1 or PD-1 inhibitor, such as nivolumab, expressing favorable effects. Taking advantage of the presence of glypican-3 on malignant hepatocytes, another vaccine has been created, the so-called glypican-3 (GPC3) vaccine. Meanwhile, the utilization of the cancer vaccine with talimogene laherparepvec and the concomitant use of ipilimumab are under evaluation in current clinical trials for either HCC or metastatic solid malignant tumors, where an intrahepatic injection is performed on the primary or secondary lesions [119,120]. Based on the fact that Alpha-fetoprotein (AFP) is highly expressed in HCC, it constitutes not only a specific biomarker for HCC diagnosis, but also a target for immunotherapy, such as in the case of AFP-based HCC vaccines that provided a weak anti-tumor effect compared to optimized AFP gene vaccine, which demonstrated an increased AFP-specific CD8 effectors’ response and it proved protective for mice against AFP positive tumor cell challenge [111,121].

### 6.4. Adaptive Cell Therapy in HCC

In this particular therapeutic modality, multiple cell types are utilized, such as cytokine-induced killer cells (CIKS), adipose- or bone-derived mesenchymal stem cells (MSCs), as well as CAR-T cells. The use of the former notably enhances overall survival, while the lattermodality exhibits a beneficial effect on limiting the escape mechanism of the tumor [122,123,124]. Some of the potential therapeutic targets exist not only in the malignant tissue but also in the physiological, such as Glypican-3 (GPC-3), which is overexpressed in HCC and infrequently in normal tissues, as well as AFP. Other targets that are present only in malignant tissues are the melanoma antigen gene family (MAGE) and New York Esophageal Squamous Cell Carcinoma-1 (NY-ESO-1). There are some other targets that are studied in basket trials, such as Claudin18.2, epithelial cell adhesion molecule (EpCAM), Epidermal growth factor receptor variant III(EGFRvIII), as well as death receptor 5 (DR5). There are ongoing trials of CAR-T cells in HCC, such as a phase I–II study (NCT03013712) that assesses EpCAM-CAR-T targeting EpCAM, aphase I study (NCT03198546) of GPC3/TGFβ-CAR-T targeting GPC3 and TGFβ in HCC, as well as a phase I study (NCT03884751) of CAR-GPC3 T Cells targeting GPC3 in HCC. Moreover, there is a basket phase I–II study (NCT03941626) of CAR-T/TCR-T cells that target EGFR vIII and DR5 [111,121].

### 6.5. Ongoing Clinical Trials and Future Approaches to HCC Management

There is an ongoing phase II study of the combinational treatment including a PD-1 inhibitor, TSR-042 (dostarlimab), with an antibody TSR-022 (Cobolimab) against T-cell immunoglobulin and mucin-domain containing-3 (TIM3) for patients with advanced HCC. TIM3 is a protein found on cytotoxic and effector T-cells that induces a defective phenotype in T cytotoxic cells. Meanwhile, there is another ongoing clinical trial using antibodies against lymphocyte activation gene-3 (LAG-3), which binds to MCH II such asCD4, inducing a suppressive effect on T cells. Antibodies against LAG-3, combined with ICIs, are considered a potential novel therapeutic strategy. Finally, there is a phase I/Ib study of NIS793 (antibody against TGF-β) plus PDR001 (spartalizumab, a PD-1 inhibitor) in patients with advanced cancers, including HCC. This potential treatment is based on the fact that TGF-β is Tregs and suppresses the effect of T-helper cells against cancer cells [106,125].

### 6.6. Predictors of Immunotherapy Response in HCC

Despite the notable clinical benefit of ICIs, only some patients actually benefit from this modality, implying that the development of predictive biomarkers is crucial for the optimal therapeutic management of HCC. There are recently reported predictive biomarkers of response to immune checkpoint blockade treatment for cases of non-resectable HCC. Examples include microsatellite instability (MSI), PD-L1, as well as tumor mutational burden (TMB). TMB constitutes the total amount of mutations that are presented in the DNA of malignant cells, which can be either 4–5 or over 10 mutations/megabases, while patients with TMB will most probably benefit from ICIs. Moreover, TMB is evaluated together with MSI as predictive biomarkers in HCC. However, only a small portion of HCC presents with high TMB and MSI-H in comparison with other malignancies, while in patients with high TMB and low MSI, the treatment response to nivolumab is optimal. The most frequently used predictive biomarker is PD-L1, which is closely associated with treatment response to either PD-L1 or PD-1 inhibition. High PD-L1 expression is associated with dismal outcomes, as well as with reduced survival in cases of advanced disease [126,127,128]. The previously neglected gut microbiome is in the spotlight, nowadays, due to the fact that is closely associated with multiple signaling and metabolic pathways, as well as with cancer development and induction of immune responses. Disruption of the microbiome is closely associated with immune resistance, which is mainly attributed to the overgrowth of Proteobacteria, whereas patients who had an increased amount of *Akkermansia muciniphila* and *Ruminococcus* spp. modulation of the microbiome in cases of dysbiosis could optimize the response to immunotherapy via fecal transplantation or concomitant administration of antibiotic therapy with ICIs, such as Vancomycin. Moreover, probiotic supplements that include Akkermansia muciniphila, Clostridium IV, and XIVa, as well as *E. faecalis* could benefit patients due to the fact that they induce suppression of species that lead to dysbiosis and finally to HCC. Gut microbiome is closely interrelated with the functional state of the liver, which is described by the gut–liver axis, via the portal circulation. The dysbiotic microbiome and the defective gut barrier lead to the production of microbial metabolites that are released into the hepatic portal circulation and subsequently influence and modify the function of bile acids (BAs). The alteration in the function of BAs is closely associated with carcinogenesis of the liver and biliary tract [111,129].

## 7. Immunotherapy in Colorectal Cancer

Colorectal cancer (CRC) constitutes the third cancer-related cause of death worldwide, with a continuously increasing trend of an occurrence arising from colon epithelium and glands, while it is estimated that cases will be further increased (>50%) by 2030. Although the mortality rate gradually decreases, the survival rate remains low and the prognosis is considered poor for disseminated metastatic disease [16].

Colorectal carcinogenesis is a multifactorial event caused by genome modifications, epigenetic aberrations, as well as the impact of environmental risk factors, which make the molecular-based therapeutic management of CRC a challenging task [130]. An important molecule that is expressed in CRCs and many other malignancies is the epidermal growth factor receptor (EGFR) [131], which can be targeted with monoclonal antibodies, such as Panitumumab and Cetuximab, without, however, showing remarkable improvement of survival and prognosis. This is a result of a gradually established resistance to these therapeutic agents correlated with certain genomic mutations of *BRAF* and *RAS* [131,132]. High microsatellite instability (MSI-H) phenotype is also observed in malignant colorectal tumors closely related to *BRAF* proto-oncogene mutation via the methylation of cytosine at the CpG islands of *BRAF* gene promoter [133]. The above phenotype is characterized by less aggressiveness and a less dismal prognosis in comparison with tumors that present microsatellite stability, while they are also associated with highly immunogenic tumor-specific antigens, the so-called neoantigens that are related to the high number of TILs in these neoplasms [134].

### 7.1. The Importance of the Tumor Microenvironment in CRC

Tumor microenvironment heterogeneity has a crucial role in CRC chemoresistance. It comprises a remarkable desmoplastic stroma that contains heterogeneous cells with a variable cellular differentiation and progression, fibroblasts, and many other immune cells, which are potential targets for anti-neoplastic agents. Cancer-Associated Fibroblasts (CAFs) are closely related to malignant tumor expansion and dissemination, while they are considered factors that contribute to poor prognosis via the secretion of cytokines that promote the proliferation of colorectal malignant cells [16]. Neoangiogenesis constitutes a vital process for the survival of cancer cells, as they develop new vessels via the effect of VEGF, provided by stromal cells, such as CAFs [135]. Tumor-associated macrophages (TAMs) also contribute to tumor invasion and metastasis, while they are sub-classified into two types: (i) anti-tumorigenic M1 and (ii) pro-tumorigenic M2 [136]. Myeloid-derived suppressor cells (MDSCs) are also present in TME in high amounts, contributing to tumor invasion and growth [137], while they are regulated by many tumor-derived substances, such as CCL5, as well as CCL2 [138].

### 7.2. Current Immune Checkpoint Inhibitors in CRC

The presence of Mismatch repair deficiency (dMMR) or Microsatellite instability-high MSI-H is associated with the deregulation of immune checkpoints molecules and with an increased mutational load. The utilization of ICIs is quite significant for this malignancy, especially for the tumors that are dMMR/MSI-H, in comparison with proficient MMR (pMMR) or microsatellite-stable (MSS) in which the response to ICIs is unfavorable [139]. The ICIs currently approved by FDA for the management of advanced metastatic dMMR/MSI-H that had undergone prior treatment with chemotherapy are ipilimumab (a CTLA-4 inhibitor), pembrolizumab (a PD-1 inhibitor), as well as nivolumab (a PD-1 inhibitor). Based on the Checkmate 142 trial, the combinational therapy of nivolumab with ipilimumab (in low dose) is considered the first line for metastatic CRC with dMMR/MSI-H. This therapeutic strategy is well tolerated and provides long-term benefits for patients who were already treated with chemotherapy including irinotecan, fluoropyrimidine, and oxaliplatin [140]. Meanwhile, in advanced metastatic CRCs, the use of pembrolizumab is approved with or without the existence of Mismatch repair deficiency (MMR) or Microsatellite instability-high MSI-H (KEYNOTE 028 clinical trial) when chemotherapeutic agents have failed [141].

### 7.3. Ongoing Trials for Cancer Vaccines in CRC

The use of cancer vaccines in CRC is currently under research, such as the Talimogene laherparepvec vaccine in cases of advanced metastatic CRC [142]. In this vaccine, a genetically modified viral vector, the oncolytic Herpes virus type 1 (HSV-1), infects the malignant tumor cells and targets the GM-CSF gene, while the use of oncolytic viruses is considered significantly potent as an anti-cancer therapy for solid malignant neoplasms [143]. A combinational treatment, including immune checkpoint blockade and cancer vaccine, is also under evaluation, such as the above vaccine with a PD-L1 inhibitor, atezolizumab, for end-stage CRC cases characterized by MSS [138]. There was a recent clinical trial (MASTERKEY-318) that evaluatedtalimogene laherparepvec vaccination as a monotherapy for solid metastatic tumors, such as in the case of secondary hepatic lesions, where intrahepatic administration is performed, while the combination of with talimogene laherparepvec vaccination treatment is also studied for HCC or metastatic hepatic disease [144].

There are various targeted antigens for CRC, such as MYB oncoproteins, which are transcription factors overexpressed in many cancers, including CRC. There are ongoing trials that assess the therapeutic or protective effects of the MYB-based DNA vaccines. Moreover, there are DC or peptide-based vaccines that are also studied in CRC. The latter category targets the tumor-associated antigens, such as MUC1, survivin, as well as CEA and signal transducer and activator of transcription 3 (STAT3); however, their application in CRC patients did not significantly increase their survival. Due to the fact that CEA is commonly found in CRC patients, the development of a targeted CEA-specific anti-cancer response is in the spotlight of many clinical studies. Moreover, a stronger immune response is induced by bacterial or viral antigen-based vaccines compared to DC and peptide-based vaccines. However, the utilization of an infectious vector could potentially be pathogenetic or mutagenic. Some examples of such vaccines are TroVax and ALVAC vaccines, with the former using vaccinia virus (attenuated strain) as a vector and the latter using a canarypox virus (non-replicating viral vector). It is reported that the combinational treatment with cytotoxic chemotherapy and ALVAC vaccine, which induces CEA- and B7-1-specific T-cell responses, is considered safe for metastatic CRC patients [145,146].

### 7.4. Future Approaches for Treating CRC

Exosome-based vaccines are considered a potential therapeutic modality, which is based on the fact that exosomes could be used as vehicles for the transport of many cargoes, such as micro RNAs (miRNAs), DNAs, and proteins, as well as messenger RNAs (mRNAs), towards the recipient cells. Exosomes constitute nanovesicles, which are usually produced by a wide variety of cells and they are closely associated with pathogenetic mechanisms, as well as carcinogenesis. Moreover, exosomes exert various effects on recipient cells through the cargoes that they transport. It has been demonstrated that exosomes produced by CRC alter the behavior of the colonic mesenchymal stromal cells, a phenomenon that may lead to the overproduction of CEA, carcinogenesis with the development of CRC, and finally metastasis. It is reported that exosomes that transport miR-20 as a cargo could lead to the dissemination of cancer cells, although exosomes could potentially be used as vehicles for the transport of therapeutic molecules, such as Heat-shock protein-70 (Hsp70), which induces an increased expression of MHCII and T-cell responses that lead to the elimination of malignant cells in murine. This implies that Hsp70 exosomes could be utilized as a novel type of vaccine for the therapeutic management of CRC [146,147,148,149]. Lastly, there are ongoing clinical trials for CAR-T cell therapies in CRC that target MUC1 (phase I/IIstudyNCT02617134), CEA (phase I study NCT03682744 and NCT02349724), NKG2DL (phase I study NCT03310008), as well as EGFR for metastatic CRC (phase I study NCT03542799), HER2 (phase I/II study NCT02713984 and phase I study NCT03740256), and CD133 (phase I/II study NCT02541370) [150].

## 8. Immunotherapy in Cholangiocarcinoma

Cholangiocarcinoma (CCA) constitutes a highly lethal entity of epithelial tumors located in the biliary tract, including three distinct heterogeneous forms, which are anatomically classified into: (i) the extrahepatic and (ii) the intrahepatic (ICC) types. ICC-type is considered the second most frequent among the primary liver cancers, followed by HCC, even though it accounts for only 10% of them. There is another rare entity of CCA that is derived from trans-differentiated hepatocytes, the so-called (CHC-CCA), whereas extrahepatic forms are the most commonly occurring, especially the perihilar type, followed by the distal type [151]. There is a global trend of continuously increasing CCA incidence presenting a notable increase in mortality rates [152,153,154]. CCA not only presents with disparities between the geographical regions, a phenomenon that is related to the environmental differences among countries, but also racial and gender particularities, demonstrating a slight male predominance (1.5-fold). In Eastern countries, the most well-studied risk factor is the consumption of contaminated food with larvae of Clonorchissinesis and Opisthorchis viverrini, as well as the exposure to the family of aflatoxins produced by Aspergillus flavus and parasiticus. In Western countries, pathologies associated with chronic biliary inflammation constitute the leading cause of CCA development, such as primary sclerosing cholangitis (PSC), non-alcoholic steatohepatitis (NASH), lithiasis of the biliary tree, as well as hepatitis C and B infection. The combinational first-choice therapy for advanced cases of CC includes cisplatin and gemcitabine [111,151,152,153]. Assessment of the effectiveness of immunotherapeutic modalities in CCA has beenin the spotlight inrecent years.

### 8.1. Immune Checkpoint Inhibitors in CCA

ICIs constitute a potential therapeutic strategy in many malignancies, including CCA. There are multiple clinical trials that assess the efficacy and safety of ICIs in CCA. Based on phase IbKEYNOTE-028 and phase II KEYNOTE-158, clinical trials that assess pembrolizumab monotherapy (a PD-1 inhibitor) for patients with advanced CCA provided a long-term anti-cancer response for cases that were non-eligible for other therapeutic options. In the former trial, pembrolizumab was given in a dose of 10 mg/kg, IV, on Day 1 of every cycle (1 cycle included 2 weeks) up to 24 months, while in the latter, it was given a dose of 200 mg IV on Day 1 of every cycle (1 cycle included 3 weeks) up to 2 years of treatment [155]. The utilization of PD-L1 inhibitors such as durvalumab is assessed in many trials (phase III TOPAZ-1 (NCT03875235)), in which the combinational treatment with durvalumab, cisplatin, and gemcitabine is compared with cisplatin, gemcitabine, and placebo as a first-line therapy for non-resectable, advanced (locally), metastatic, or recurrent CCA that was treatment-naive. The experimental treatment arm includes the IV administration of durvalumab 1500 mg every 3 weeks with gemcitabine (1000 mg/m^2^) plus cisplatin (25 mg/m^2^) on Days 1 and 8, every 3 weeks up to 8 cycles, followed by durvalumab monotherapy, 1500 mg every 4 weeks until either the appearance of severe toxicity or disease advancement, while the placebo arm includes placebo IV infusion every 3 weeks with gemcitabine (1000 mg/m^2^) plus cisplatin (25 mg/m^2^) on Days 1 and 8, every 3 weeks up to 8 cycles, followed by (placebo) monotherapy every 4 weeks similarly until the appearance of discontinuation criteria or disease advancement [156].The results of the aforementioned trial for durvalumab as a single-agent treatment or in combination are still expected. Moreover, there is a phase II (NCT02829918) study of nivolumab (a PD-L1) for advanced cases of biliary tract malignancy in an IV dose of 240 mg every 2 weeks up to 16 weeks, followed by 480 mg every 4 weeks until disease advancement or unacceptable toxicity. In the aforementioned trial, nivolumab demonstrated a moderate durable efficacy in refractory cases of biliary tract cancer [157]. Furthermore, there is a phase II trial IMbrave [151] that assesses atezolizumab with bevacizumab (VEGF inhibitor) on a chemotherapy backbone of Gemcitabine and Cisplatin for treating advanced CCA. Based on an ongoing phase I trial (NCT03101488), a partial response (PR) was observed for twopatients with biliary tract malignancy that received the novel subcutaneously injected anti-PD-L1 antibody, Envafolimab (KN035) [158]. In Table 2 we present a summary of the results from clinical trials that assess the utilization of ICIs in CCA management.

### 8.2. Cell-Based Therapies and Cancer Vaccines for CCA

Another modality, based on the CAR-T cells against molecular targets on the cancer cell surface, such as epidermal growth factor receptor (EGFR), and CD133 is still under assessment. Although they are promising as an anti-neoplastic modality, their use is limited due to the abundance of adverse effects. Similar to the HCC-cancer vaccines, there are vaccines also applied in CCA cases, such as peptide and DC-based vaccines; however, with limited therapeutic effects. Some of the tumor-associated antigens (TAAs) that could be potential targets for peptide vaccines in CCA are MUC1 and Wilms’ tumor protein 1 (WT1). Finally, there are ongoing studies of Tumor lysate-based DC vaccines, which demonstrated an early invitro efficacy, while MUC1-loaded DC vaccine used as adjuvant treatment after the resection of biliary tract and pancreatic malignancies did not exert anti-MUC-1 antibody responses [159,160].

### 8.3. Future Approaches for Treating CCA

Future approaches for the optimal treatment of CCA include the identification of predictive biomarkers for durvalumab response in CCA, based on TMB, MSI/MMR, and PD-L1, such as in case of HCC [161]. Additionally, there is a promising clinical trial (NCT04910386) that has not yet recruited patients that is going to assess the combinational treatment of Envafolimab with gemcitabine and cisplatin, in comparison with gemcitabine and Cisplatin as a first-line therapy for cases of either metastatic or locally advanced CCA [162]. Toripalimab constitutes another PD-1/PD-L1 ICI, which will be assessed in an ongoing trial (NCT03867370 study) as a neoadjuvant treatment combined with Lenvatinib for resectable HCC, including the intrahepatic CCA [163].

Meanwhile, the recognition and development of personalized targets (tumor-associated antigens) might improve the clinical efficacy of T-cell therapies or cancer vaccines in CCA, while ICI-centered combinations, as well as individualized therapy (TT) will be the focus of research for CCA management [164].

In Table 3, we provide a summarized table with immune checkpoint inhibitors, cancer vaccines, oncolytic viral therapies, and adoptive cell therapies for gastrointestinal cancers.

## 9. The Pros and Cons of the Immunotherapeutic Modalities

Immunotherapy elicits an immune-inflammatory effect that has high accuracy and specificity towards the targeted tissue. It provides a durable effect as it stimulates the immune system of the patient to start killing the cancer cells in the long term. Moreover, it restores the immune system of the patient and can potentially prevent cancer recurrence and metastasis. On the other hand, one of the main disadvantages of immunotherapy is the fact that this modality needs to be precisely selected for the patients based on the type of their tumors. Although ICIs provide favorable effects for many malignancies, they can induce various side effects; they are costly and sometimes lead to autoimmune diseases or even death, due to the aggravation of the general status of the patients. However, the adverse effects of immunotherapy are still considered less in comparison with chemotherapy. Some adverse effects that manifest during the utilization of immunotherapeutic agents are skin toxicity rash and pruritus up to severe manifestations such as toxic epidermal necrolysis (TEN), Steven-Johnson syndrome, and Sweet syndrome in cases of ICIs toxicity. There are cases of PD-1/PD-L1 associated adverse effects, such as immune-related pneumonitis, as well as acute respiratory distress syndrome (ARDS) and diffuse alveolar damage. The most common side effects of ICIs are gastrointestinal disruptions, such as enterocolitis after CTLA-4 inhibitors, in comparison with PD-1 inhibitors, which usually induce acute colonic pseudo-obstruction. There are reports of disruption of the liver function tests during the use of these agents. However, in every patient with hepatotoxicity, viral hepatitis must be necessarily excluded. Last but not least, there are cases of endocrinological side effects, such as thyroid dysfunction and even severe cases of hypophysitis after the utilization of CTL4-inhibitors and de novo diabetes mellitus after PD-L1/PD-1 blockade [165,166].

## 10. Conclusions

A continuously increasing number of GI cancer cases constitutes a major cause of cancer-related deaths globally. The multifactorial epidemiologic background of these malignancies and the wide variety of genomic and epigenetic modifications open new therapeutic opportunities for their management. Immunotherapy is under the focus of studies, including a wide spectrum of agents such as cancer vaccines, ACT therapy, and immune checkpoint inhibitors, which constitute alternative therapeutic strategies when conventional chemotherapy has failed, or they take part in enhancing the anti-cancer effect of other methods. The specificity of this strategy includes its direct effect on cancer cells, which makes it less toxic compared to conventional treatments. Nevertheless, the application of immunotherapy is mainly limited to advanced cancer, when chemotherapeutic agents have proved inefficient, while its combination with other treatment modalities demonstrates promising results with significantly improved survival and prognosis. However, further investigation is required to develop immunotherapeutic agents that could benefit patients as monotherapy or combinational treatment in early or advanced stages of GI carcinogenesis.

## Figures and Tables

**Table 1:** Summary of the results from clinical trials in HCC with ICI.

Clinical Trial	Drug	Phase	Results
Oriental	Sorafenib	Phase III, randomized, double-blind, placebo-controlled	6.5 vs. 4.2 months OS 2.8 vs. 1.4 months TTP
Sharp	Sorafenib	Phase III Randomized, double-blind, Placebo-controlled	10.7 vs. 7.9 months OS 5.5 vs. 2.8 months TTP 43% vs. 32% DCR
Reflect	Lenvatinib vs. sorafenib	Phase III, open-label, multicenter, non-inferiority	13.6 vs. 12.3 months OS 7.4 vs. 3.7 months TTP
CheckMate 459	Nivolumab vs. sorafenib	Phase III, randomized, open-label	16.4 vs. 14.7 months OS
KEYNOTE-224	Pembrolizumab	Phase II, non-randomized, multicenter, open-label	13.2 months OS 4.8 months TTP 61.5% DCR
IMbrave150	Atezolizumabplusbevacizumabvssorafenib	Phase III study, randomized, open-label	19.2 vs. 13.4 months OS 6.9 vs. 4.3 months PFS

Overall survival (OS); time to progression (TTP); disease control rate (DCR); progressive-free survival (PFS).

Overall survival (OS); progressive-free survival (PFS); objective response rate (ORR).

**Table 2:** Summary of the results from clinical trials in CCA with ICI.

Clinical Trial	Regimen	Phase	Results
MSB0011359C (M7824) in Subjects With Metastatic or Locally Advanced Solid Tumors	Bintrafuspalfa	Phase I, open-label trial expansion cohort	12.7 months OS 2.5 months PFS 20% ORR
TOPAZ-1	Durvalumab plus gemcitabine and cisplatin vs. gemcitabine and cisplatin	Phase III, randomized, double-blinded clinical trial	12.8 vs. 11.5 months OS 7.2 vs. 5.7 months PFS 26.7 vs. 18.7 months ORR
INTR@PID BTC 055	Bintrafuspalfa plus gemcitabine and cisplatin	Phase II, open-label, randomized, double-blinded	10.1% ORR
IMMUNOBIL PRODIGE 57	Durvalumab and tremelimumab vs. durvalumabplustremelimumab and paclitaxel	Phase II, non-comparative randomized	Raising safety concerns regarding co-administration of paclitaxel with durvalumab and tremelimumab
KEYNOTE-158	Pembrolizumab	Phase II, non-randomized, open-label	23.5 months OS 4.1 months PFS 34.3 ORR
A Phase 2 Clinical Trial of Entinostat in Combination With Nivolumab for Patients With Previously Treated Unresectable or Metastatic Cholangiocarcinoma and Pancreatic Adenocarcinoma	Entinostat plus nivolumab	Phase II, open-label	6.4 months OS
A Randomized Phase 2 Study of Atezolizumab in Combination With Cobimetinib Versus Atezolizumab Monotherapy in Participants With Unresectable Cholangiocarcinoma	Atezolizumab vs. Atezolizumabpluscobimetinib	Phase II, open-label randomized	3.65 vs. 1.87 months PFS
CA209-538	Nivolumab and ipilimumab	Phase II, non-randomized	5.7 months OS 2.9 months PFS 23% ORR

Overall survival (OS); progressive-free survival (PFS); objective response rate (ORR).

**Table 3:** Summary of immunotherapy for gastrointestinal cancers.

Immunotherapy Modality.	Agents
**Immune checkpoint inhibitors**	
Pancreatic cancer	
PD-1 inhibitors	Nivolumab, Pembrolizumab
PD-L1 inhibitors	
CTLA-4	Tremelimumab, Ipillimumab
**Gastric cancer**	
PD-1 inhibitors	Nivolumab, Pembrolizumab
PD-L1 inhibitors	Atezolizumab
CTLA-4	
**Hepatocellular carcinoma**	
PD-1 inhibitors	Nivolumab, Pembrolizumab
PD-L1 inhibitors	Atezolizumab
CTLA-4	
**Colorectal cancer**	
PD-1 inhibitors	Nivolumab, Pembrolizumab
PD-L1 inhibitors	
CTLA-4	Ipillimumab
**Cholangiocarcinoma**	
PD-1 inhibitors	Nivolumab, Pembrolizumab, Bintrafuspalfa
PD-L1 inhibitors	Durvalumab
CTLA-4	Tremelimumab, Ipillimumab
**Cancer vaccines**	
**Pancreatic cancer**	Gvax, Peptide vaccines, mKras vaccine, CV301, GI-4000
**Gastric cancer**	PAS-vaccination
**Hepatocellular carcinoma**	HEPAVAC, dendritic cell vaccine (DC vaccine), glypican-3 (GPC3) vaccine
**Colorectal cancer**	Talimogene laherparepvec vaccine
**Cholangiocarcinoma**	DC-based vaccines
**Oncolytic viral therapy**	
**Pancreatic cancer**	Adeno-associated viruses (AVV), Herpes Simplex Virus-1 and 2 (HSV-1 and HSV-2), HSV1716, R3616, vaccinia virus, rabbit-MYXV poxvirus
**Hepatocellular carcinoma**	HSV-1-based, adenovirus-based (CNHK500, ONYX-015, AD, ZD55-IFN-β, Smac/ZD55-TRAIL), vaccinia-based (JX-594 therapy)
**Adaptive cell therapy**	
**Pancreatic cancer**	Targets: MUC1, mesothelin, and CEA, FAP, HER2, PSCA, CD24,
**Hepatocellular carcinoma**	cytokine-induced killer cells (CIKS), bone-derived mesenchymal stem cells (MSCs), CAR-T cell treatment
**Colorectal cancer**	CAR-T cells treatment
**Cholangiocarcinoma**	CAR-T cells against epidermal growth factor receptor (EGFR), and CD133

## Data Availability

Not applicable.

## Abbreviations

(CB1)/(CB2)	cannabinoid receptor 1 and receptor2
(ceRNA)	competing endogenous RNA
(CMA)	chaperon-mediated autophagy
(HCQ)	hydroxychloroquine
(HIF-1a)	hypoxia-inducible factor-1
(HMGB1)	high-mobility group box 1
(LAMP-2A)	lysosomal membrane protein 2A
(lncRNA)	long noncoding RNA
(microRNAs)	small non-coding RNA molecules
(MSI-H)	microsatellite instability
(mTOR)	mechanistic target of rapamycin
(PADC)	pancreatic adenocarcinoma
(PE)	lipid phosphatidylethanolamine
(PVT1)	plasmacytoma variant translocation 1
(Pygo2)	pygopus2
(RAGE)	receptors for advanced glycation end products
(ROS)	reactive oxygen species
(siRNA)	small interfering RNA
(TME)	tumor microenvironment
(UA)	ursolic acid
(ULK1)	Unc-51-like kinase1 complex
(VMP1)	vacuole membrane protein 1
